# *In vitro* effects of histamine receptor 1 antagonists on proliferation and histamine release in canine neoplastic mast cells

**DOI:** 10.1002/vms3.336

**Published:** 2020-09-13

**Authors:** Susanne Gamperl, Gabriele Stefanzl, Michael Willmann, Peter Valent, Emir Hadzijusufovic

**Affiliations:** ^1^ Division of Hematology & Hemostaseology Department of Internal Medicine I Medical University of Vienna Vienna Austria; ^2^ Department of Companion Animals and Horses Clinic for Internal Medicine and Infectious Diseases University of Veterinary Medicine Vienna Vienna Austria; ^3^ Ludwig Boltzmann Institute for Hematology and Oncology Medical University of Vienna Vienna Austria

**Keywords:** histamine release, HR1 antagonists, mast cell, MCT

## Abstract

Canine mastocytomas (MCTs) are characterized by rapid proliferation of neoplastic mast cells (MCs) and clinical signs caused by MC‐derived mediators. In dogs suffering from MCT, histamine receptor 1 (HR1) antagonists are frequently used to control mediator‐related clinical symptoms. Previous studies have shown that the HR1 antagonists loratadine and terfenadine exert some growth‐inhibitory effects on neoplastic MCs. We examined whether other HR1 antagonists used in clinical practice (desloratadine, rupatadine, cyproheptadine, dimetindene, diphenhydramine) affect proliferation and survival of neoplastic MCs. Furthermore, we analysed whether these HR1 antagonists counteract IgE‐dependent histamine release from a MC line harbouring a functional IgE‐receptor. HR1 antagonists were applied on two canine MC lines, C2 and NI‐1, and on primary MCs obtained from three MCT samples. The HR1 antagonists desloratadine, rupatadine and cyproheptadine were found to be more potent in decreasing proliferation of C2 and NI‐1 cells when compared with dimetindene and diphenhydramine. Similar effects were seen in primary neoplastic MCs, except for diphenhydramine, which exerted more potent growth‐inhibitory effects than the other HR1 antagonists. Drug‐induced growth‐inhibition in C2 and NI‐1 cells was accompanied by apoptosis. Loratadine, desloratadine and rupatadine also suppressed IgE‐dependent histamine release in NI‐1 cells. However, drug concentrations required to elicit substantial effects on growth or histamine release were relatively high (>10 µM). Therefore, it remains unknown whether these drugs or similar, more potent, HR1‐targeting drugs can suppress growth or activation of canine neoplastic MCs *in vivo*.

## Graphical abstract

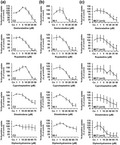

## 1 INTRODUCTION

Mastocytomas (MCT) are one of the most frequent malignant skin neoplasms in dogs, ranging from well differentiated tumours often curable by surgery to rapidly proliferating aggressive variants with short survival times (Bostock, 1986; Brodey, 1970; Finnie & Bostock, 1979; Rothwell, Howlett, Middleton, Griffiths, & Duff, 1987). Clinical symptoms result from organ damage due to the accumulation of neoplastic mast cells (MCs) and/or effects of proinflammatory MC‐derived mediators, such as histamine (Misdorp, 2004). These manifestations are similar to clinical signs in human patients suffering from MC neoplasms, such as systemic mastocytosis (Valent et al., 2003). Mediator‐related clinical symptoms in dogs can be mild, but can also lead to serious reactions (Thamm & Vail, 2007; Blackwood et al., 2012), such as anaphylaxis comparable to human patients (Valent et al., 2003). In both species, an established approach to counteract mediator‐related clinical symptoms is to treat the patients with histamine receptor 1 (HR1) antagonists (Akin & Metcalfe, 2004; Eichenseer, Johansen, & Mueller, 2013; Metcalfe, 2008; Pariser & Gram, 2015; Peters & Kovacic, 2009; Welle, Bley, Howard, & Rufenacht, 2008).

Several previous studies have shown that HR1 antagonists exert growth‐inhibitory effects on different tumour cells *in vitro*, including melanoma cells and myeloid leukaemia cells (Aichberger et al., 2006; Jangi et al., 2006). In addition, it has been described that the HR1 antagonists terfenadine and loratadine inhibit spontaneous growth of human, feline and canine neoplastic MCs (Hadzijusufovic et al., 2010). However, terfenadine has been removed from the market and loratadine inhibits growth and survival of MCs only at higher concentrations.

Therefore, we were interested to test other HR1 antagonists used either in veterinary medicine (diphenhydramine) (Peters & Kovacic, 2009), or in human medicine (desloratadine, rupatadine, dimetindene, cyproheptadine) (Gimenez‐Arnau, Izquierdo, & Maurer, 2009; Gunja, Collins, & Graudins, 2004; Horak, Unkauf, Beckers, & Mittermaier, 2011; Siebenhaar, Degener, Zuberbier, Martus, & Maurer, 2009) for their anti‐neoplastic effects on canine neoplastic MCs. In addition, loratadine, the prodrug for desloratadine, was used as positive control. In this study we also tested multi‐targeted tyrosine kinase inhibitors (TKIs), such as masitinib, toceranib and midostaurin, which are currently used to treat canine or human neoplastic MC disorders. Masitinib and toceranib are approved to treat recurrent or non‐resectable high‐grade MCT with or without regional lymph node involvement in dogs, whereas midostaurin has recently been approved for treatment of human patients with advanced systemic mastocytosis, including mast cell leukaemia (Gleixner et al., 2013; Gotlib et al., 2016; Hahn et al., 2008; London, 2009; London et al., 2009). We were interested to learn whether combinations of HR1 antagonists and TKIs could lead to cooperative drug effects and thus a reduction in the dose of the individual drug. Indeed, such an approach has also been followed in previous studies where different TKIs were combined with each other and were found to exert additive or synergistic anti‐proliferative effects in human or canine neoplastic MCs (Gleixner et al., 2007, 2013). In our studies we used *in vitro* methods employing two established canine MC lines, C2 and NI‐1 (DeVinney & Gold, 1990; Hadzijusufovic et al., 2012) and primary MCs isolated from three MCT samples. We tested drug effects on proliferation and survival of neoplastic MCs as well as on IgE‐dependent histamine release from NI‐1 cells, known to express a functional IgE‐receptor (Hadzijusufovic et al., 2012). C2 cells could not employed in these experiments due to their lack of a functional IgE‐receptor (Brazís et al., 2002).

The aim of our study was to define growth‐inhibitory and histamine release‐suppressing effects of various HR1 antagonists in neoplastic canine MCs and to identify the most effective HR1‐targeting drugs that could be further tested in clinical studies.

## 2 MATERIALS AND METHODS

### 2.1 Drugs and reagents

Detailed characteristics of HR1 antagonists and TKIs used in this study are shown in Table 1. Stock solution of cyproheptadine was prepared by dissolving in 100% ethanol (Merck, Darmstadt, Germany). Stock solutions of all other drugs were prepared by dissolving in dimethylsulphoxide (DMSO; Sigma‐Aldrich, St. Louis, MO, USA). RPMI 1,640 medium and antibiotics (penicillin/streptomycin) were purchased from Lonza (Basel, Switzerland). Amphotericin B was purchased from PAN‐Biotech (Aidenbach, Germany). Foetal bovine serum (FBS) was purchased from Gibco Life Technologies (Carlsbad, CA, USA) and ^3^H‐thymidine was obtained from PerkinElmer (Waltham, MA, USA).

**TABLE 1:** Histamine receptor 1 (HR1) antagonists and tyrosine kinase inhibitors (TKIs) used in this study

	Name	Generation	Known targets	Clinical application	C_max_ in µM (at dose/day)^a^	Applied concentration range (µM)^b^	Supplier^c^
**Histamine receptor 1 antagonists**	Cyproheptadine	1st	H_1_ receptor	human: hay fever	0.1 (8 mg) Gunja et al. (2004)	0.1–55	Selleck Chemicals
	Dimetindene	1st	H_1_ receptor, CHRM2	human: allergic reactions	0.02 (4 mg) Kauert, Herrle, and Wermeille (1993)	0.1–75	Sigma‐Aldrich
	Diphenhydramine	1st	H_1_ receptor	human: allergic reactions	0.06 (25 mg) Valoti, Frosini, Dragoni, Fusi, and Sgaragli (2003)	0.1–75	Sigma‐Aldrich
	Loratadine	2nd	H_1_ receptor	human: AR, hay fever and CIU	0.01 (10 mg) Salmun et al. (2000)	0.1–50	Sigma‐Aldrich
	Desloratadine	2nd	H_1_ receptor	human: AR, hay fever and CIU	0.01 (5 mg) Affrime, Gupta, Banfield, and Cohen (2002); Gupta et al. (2002)	0.1–50	Sigma‐Aldrich
	Rupatadine	2nd	H_1_ receptor, PAFR	human: AR and CIU	0.01 (10 mg) Taubel et al. (2016)	0.1–50	Sigma‐Aldrich
**Tyrosine kinase inhibitors**	Masitinib	—	KIT, PDGFR, LCK, LYN, FGFR3	canine: MCT	1.1 (12.5 mg/kg) Hahn et al. (2008)	10	LC Laboratories
	Midostaurin	—	KIT, PKC, PKA, S6, EGFR, PDGFR	human: AML, mastocytosis, MCL	7 (225 mg) Stone et al. (2005)	0.009–10	LC Laboratories
	Toceranib	—	KIT, VEGFR, PDGFR, CSF‐1, FLT3	canine: MCT	0.3 (3.25 mg/kg) Yancey, Merritt, Lesman, Boucher, and Michels (2010)	10	Sigma‐Aldrich

Abbreviations: AML, acute myeloid leukaemia; AR, allergic rhinitis; CHRM2, cholinergic receptor, muscarinic 2; CIU, chronic idiopathic urticaria; C_max_, peak plasma concentration of a drug after administration; CSF‐1, colony‐stimulating factor 1; EGFR, epidermal growth factor receptor; FGFR3, fibroblast growth factor receptor 3; FLT3, fms like tyrosine kinase 3; KIT, tyrosine‐protein kinase c‐Kit; LCK, leucocyte C‐terminal SRC kinase; LYN, LCK/YES novel tyrosine kinase; MCT, mast cell tumour; PAFR, platelet‐activating factor receptor; PDGFR, platelet‐derived growth factor receptor; PK, protein kinase; VEGFR, vascular endothelial growth factor receptor.

^a^ C_max_ values indicate human peak plasma concentrations, except for masitinib and toceranib, which were measured in dogs

^b^ Range of concentrations used in this study.

^c^ LC Laboratories, Woburn, MA, USA; Selleck Chemicals, Houston, TX, USA; Sigma‐Aldrich, St. Louis, MO, USA

### 2.2 Cell lines and culture conditions

Two canine mastocytoma cell lines were used: C2 and NI‐1. C2 cells were kindly provided by Dr. Warren Gold (Cardiovascular Research Institute, University of California, San Francisco, CA, USA) (DeVinney & Gold, 1990). NI‐1 cells, exhibiting a functional IgE receptor, were established in our laboratory in 2012 (Hadzijusufovic et al., 2012). Both cell lines were cultured in RPMI 1,640 medium containing 10% FBS, 1% antibiotics (penicillin/streptomycin) and 1% amphotericin B at 5% CO_2_ and 37°C and 95% relative humidity. Cells were thawed from an original stock, kept in culture for 6–8 weeks and split every 2–3 days. We confirmed the identity of our cell lines in regular intervals by morphologic and phenotypic characterization. Mycoplasma negativity of cell lines was confirmed regularly once a month. In brief, genomic DNA was extracted from cell lines (1 × 10^6^ cells) using the QIAmp DNA Mini isolation kit (Qiagen, Venlo, Netherlands) and tested for mycoplasma using the Venor GeM Classic, Mycoplasma Detection Kit for Conventional PCR (Minerva Biolabs Inc., Hillsborough, NJ, USA) according to the manufacturers’ instructions. DMSO and ethanol were applied as vehicle controls (in concentrations corresponding to the highest drug concentrations). These vehicle controls showed no effects (Figure S1).

### 2.3 Isolation of primary canine neoplastic MCs from mastocytoma specimens

Fresh MCT samples were obtained from three dogs undergoing surgery at the University of Veterinary Medicine Vienna (Vienna, Austria). Detailed characteristics of mastocytoma patients are listed in Table 2. Primary MCs were isolated using collagenase digestion as essentially described (Valent et al., 1989). In particular, tissue samples were cut into small pieces, washed thoroughly in Ca^2+^/Mg^2+^ free Tyrode's buffer and were then incubated in 75 mg collagenase type 2 (330 U/mg; Worthington Biochemical Corporation, Lakewood, NJ, USA) dissolved in 50 ml 0.9% NaCl at 37°C for 3 hr. Collagenase activity was stopped by adding RPMI 1,640 medium with 10% FBS. Thereafter, cells were centrifuged, and the supernatant discarded. Isolated MCs were washed with Ca^2+^/Mg^2+^ free Tyrode's buffer, then recovered by filtration through a cell strainer (70 µM pore size) and collected in RPMI medium with 10% FBS. Cells were examined for viability by trypan blue exclusion and for the percentage of MCs by Wright Giemsa staining. Isolated MCs were used for ^3^H‐thymidine uptake experiments on the day of isolation.

**TABLE 2:** Canine mastocytoma patients’ characteristics and response of neoplastic cells to histamine receptor 1 (HR1) antagonists

No (#)	Sex (f/m)	Age (years)	Breed	Histologic Grade^a^	IC_50_ ranges^c^ (µM) obtained with					
					Loratadine^b^	Desloratadine	Rupatadine	Cyproheptadine	Dimetindene	Diphenhydramine
1	fs	5.3	Magyar Vizsla	I	10–20	20–35	20–35	35–50	>75	10–25
2	mc	9	American Staffordshire Terrier	III	5–10	20–35	10–20	>50	25–50	10–25
3	fs	5.9	Mixed Breed	II‐III	20–35	20–35	35–50	20–35	25–50	10–25

Abbreviations: fs, female spayed; IC, inhibitory concentration; mc, male castrated; No, number.

^a^ Histologic Grade according to the Patnaik, Ehler, and MacEwen (1984) scheme.

^b^ Serves as positive control.

^c^ Assessed by ^3^H‐thymidine uptake after 64 hr.

### 2.4 Measurement of proliferation of canine neoplastic MCs after incubation with drugs

Cells were seeded in 96‐well microtiter plates (5 × 10^3^ C2 or NI‐1 cells/well; 15 × 10^4^ primary MCs/well) and incubated in control medium, vehicle control or various concentrations of HR1 antagonists (0.1–75 µM) at 37°C for 48 hr. Since the concentrations of HR1 antagonists required to block MCT cell growth were rather high, we were interested to identify drug‐combinations in which the concentrations of the individual drugs could be reduced to a pharmacologically meaningful range. Therefore, we tested combinations of HR1 antagonists and TKIs at suboptimal concentrations (20%–30% maximal proliferation reduction) to evaluate cooperative effects. After incubation, 0.5 µCi of ^3^H‐thymidine was added (37°C, 16 hr). Thereafter, cells were harvested on filter membranes (Perkin Elmer, Waltham, MA, USA) in a Filtermate 196 harvester (Packard Bioscience, Meriden, CT, USA) and the bound radioactivity was measured in a MicroBeta^2^ Counter (Perkin Elmer). All experiments were performed in triplicates and experiments with cell lines were repeated at least three times.

### 2.5 Evaluation of apoptosis in drug exposed cells and confirmation by TUNEL assay

C2 and NI‐1 cells were incubated in control medium, vehicle control or in various concentrations of HR1 antagonists (30–125 µM) in 24‐well microtiter plates (5 × 10^5^ cells/well) at 37°C for 24 or 48 hr. After incubation, cells were harvested and cytospin slides were prepared using Cytospin^TM^ 4 Cytocentrifuge from ThermoFisher Scientific (Waltham, MA, USA). Cytospin slides were stained with the Hematek® Stain Pak (Modified Wright's Stain #10310965) obtained from Siemens Healthineers (Erlangen, Germany) on a Hematek® Slide Stainer obtained from Bayer HealthCare LLC (Leverkusen, Germany) and three visual fields per cytospin slide were quantified by light microscopy. In three visual fields (400× magnification), approximately 200 cells were counted and the percentages of viable, apoptotic and necrotic cells were calculated from total cell numbers. Apoptosis and necrosis were defined according to characteristic morphological features (Van Cruchten & Van Den Broeck, 2002). Apoptosis in drug‐exposed cells was confirmed by the “TUNEL‐In situ cell death detection kit‐fluorescein” kit from Roche (Mannheim, Germany) following the manufacturer's instructions. The fluorescent stain 4′,6‐diamidino‐2‐phenylindole (DAPI; Sigma‐Aldrich) was used to visualize cell nuclei. Images were obtained using Zen imaging software and Axio Imager 1, both from Carl Zeiss (Oberkochen, Germany).

### 2.6 Histamine release experiments

For sensitization, NI‐1 cells were pre‐loaded with 5 µg/ml purified dog IgE (P015; Bethyl Laboratories, Montgomery, TX, USA) at 37°C for 2 hr. Thereafter, the cells were incubated with HR1 antagonists alone or in combination with TKIs at various concentrations at 37°C for 60 min. Afterwards, cells were washed in phosphate‐buffered saline (PBS; Gibco Life Technologies), resuspended in histamine release buffer (HRB; Immunotech, Marseille, France) and then incubated with 5 µg/ml goat anti‐dog‐IgE antibody (A40‐125A; Bethyl Laboratories) at 37°C for 30 min. After incubation, cells were centrifuged at 4°C, and the cell‐free supernatants and cell pellets were recovered and analysed for histamine content by radioimmunoassay (RIA; Immunotech). Total histamine (cellular + extracellular) was measured in lysates of non‐sensitized NI‐1 cells. Histamine release was calculated as the amount of histamine in the supernatant and expressed as percentage of total histamine. All experiments were performed in triplicates.

### 2.7 Statistical analysis

Statistical significance of results was determined using the Student's *t* test for independent samples or ANOVA with Bonferroni–Holm correction for dose–response experiments. Results were considered statistically significant when *p* was < .05 (Asterisk). Combination index (CI) values for drug combinations in histamine release experiments were determined using Calcusyn (Biosoft, Ferguson, MO, USA) and considered to be synergistic when the CI was <1, additive when CI = 1 and antagonistic when CI > 1.

## 3 RESULTS

### 3.1 Effects of HR1 antagonists on proliferation of canine MC lines

**FIGURE 1:**
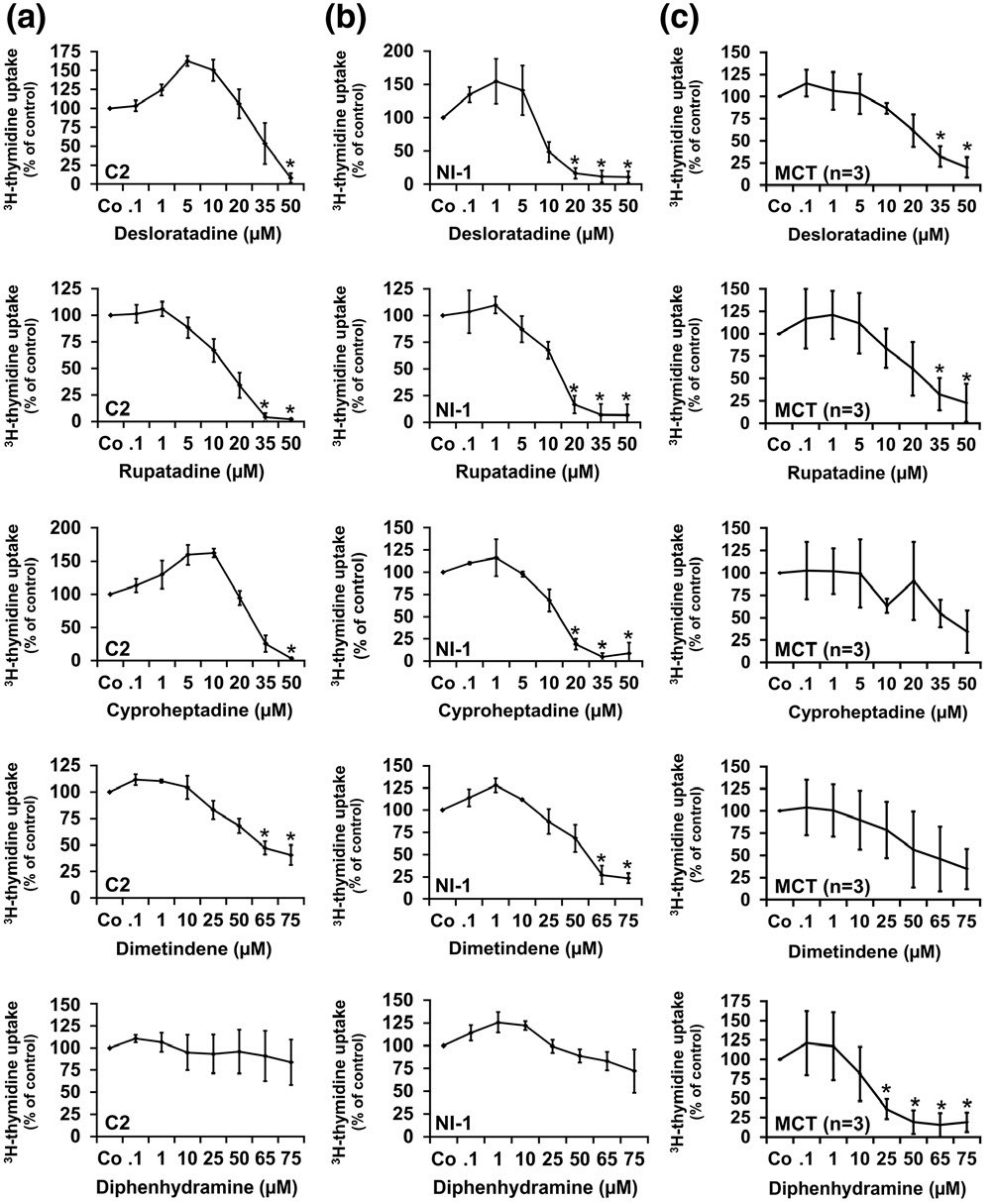
Effects of HR1 antagonists on proliferation of canine neoplastic MCs. C2 cells (a), NI‐1 cells (b) and three primary MCT cells (c) were incubated in control medium (Co) or in medium containing various concentrations of HR1 antagonists (as indicated) at 37°C for 48 hr. Thereafter, ^3^H‐thymidine was added for 16 hr and then the uptake of ^3^H‐thymidine was measured. Results show ^3^H‐thymidine uptake in percent of control (=100%, Co) and represent the mean ± *SD* of at least three independent experiments. Asterisk (*): *p* < .05 compared with control (Co)

As assessed by ^3^H‐thymidine uptake experiments, various HR1 blockers suppressed proliferation of C2 and NI‐1 cells as well as of primary MCs. In MC lines, the following rank order of potency was found: desloratadine > rupatadine > cyproheptadine > dimetindene > diphenhydramine (Figure 1a,b). In primary MCs (*n* = 3), HR1 antagonists reduced proliferation according to following rank order of potency: diphenhydramine > desloratadine > rupatadine > cyproheptadine > dimetindene (Figure 1c). Responses of MCT cells to these drugs in the individual samples (patients, #1–3) are shown in the Supplementary Material (Figure S2). Diphenhydramine was found to exert more potent growth‐inhibitory effects in primary MCs compared with MC lines (Figure 1, Tables 2 and 3). IC_50_ ranges are shown in Table 2 (primary MCs) and Table 3 (MC lines). Furthermore, HR1 antagonists were found to be more potent against NI‐1 cells compared with C2 cells (Figure 1, Table 3).

**TABLE 3:** Effects of histamine receptor 1 (HR1) antagonists on proliferation/survival of canine neoplastic mast cells

	IC_50_ ranges^a^ (µM) obtained with					
	Loratadine^b^	Desloratadine	Rupatadine	Cyproheptadine	Dimetindene	Diphenhydramine
**C2**	5–10	20–35	10–20	20–35	50–65	>75
**NI−1**	5–10	5–10	10–20	10–20	50–65	>75

	ED_50_ ranges^c^ (µM) obtained with					
	Loratadine^b^	Desloratadine	Rupatadine	Cyproheptadine	Dimetindene	Diphenhydramine
**C2**	15–25	30–35	40–45	40–50	100–125	>125
**NI−1**	15–25	22.5–25	25–30	30–35	>100	>100

Abbreviations: ED, effective dose; IC, inhibitory concentration.

^a^ assessed by ^3^H‐thymidine uptake after 64 hr.

^b^ Serves as positive control.

^c^ Assessed by morphological evaluation after 48 hr.

### 3.2 Effects of HR1 antagonists on survival of canine MC lines

**FIGURE 2:**
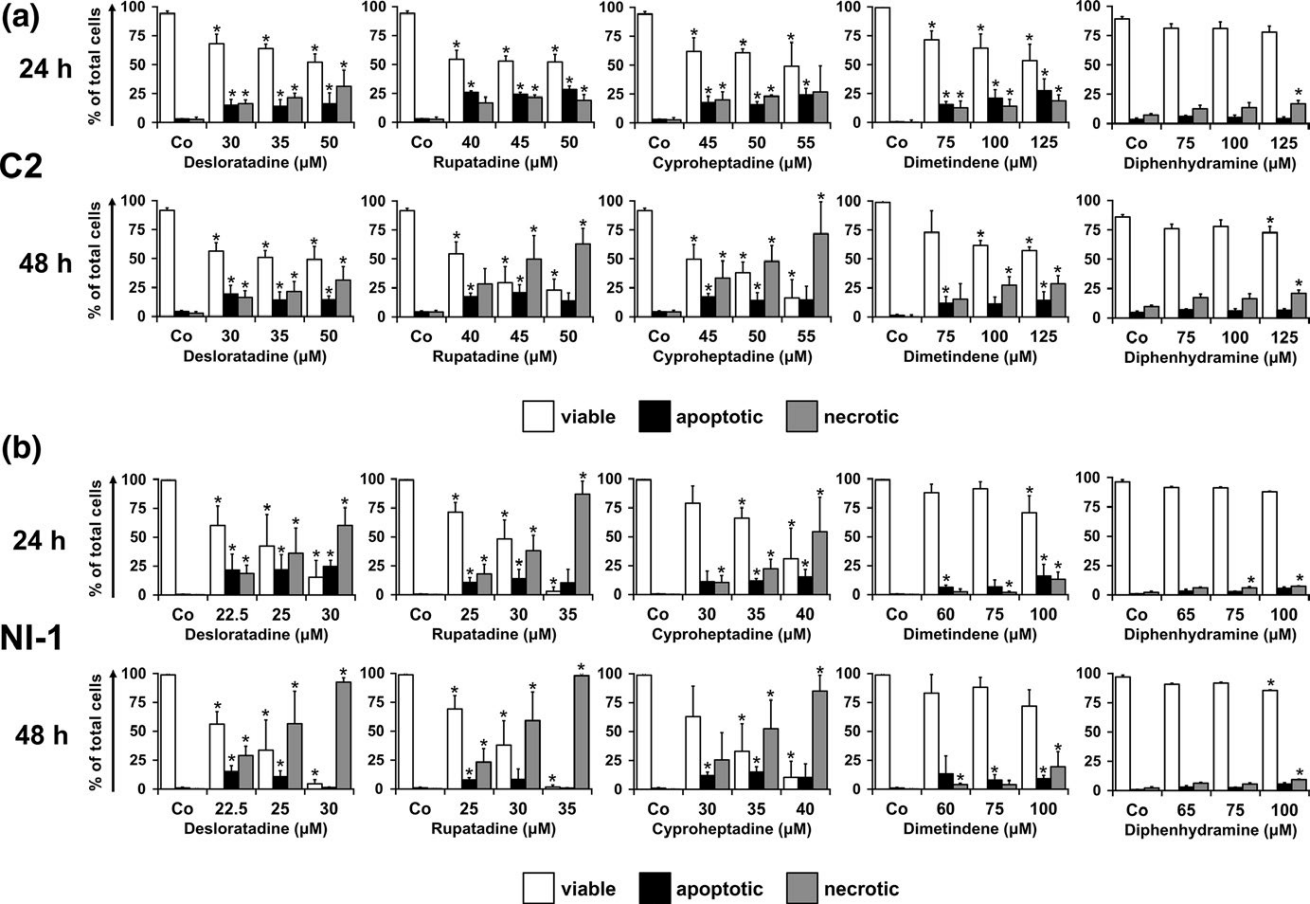
Induction of apoptosis by HR1 antagonists in C2 cells and NI‐1 cells. C2 cells (a) and NI‐1 cells (b) were incubated in control medium (Co) or in medium containing various concentrations of HR1 antagonists (as indicated) at 37°C for 24 hr (upper panels) or 48 hr (lower panels). Thereafter, the cells were stained using the Hematek® Stain Pak (Modified Wright's Stain) and the numbers of viable, apoptotic and necrotic cells were counted using light microscopy. Results show the percentage (%) of viable (white open bars), apoptotic (black filled bars) and necrotic (grey filled bars) cells relative to the total cell number. Results represent the mean ± *SD* of at least three independent experiments. Asterisk (*): *p* < .05 compared with control (Co)

**FIGURE 3:**
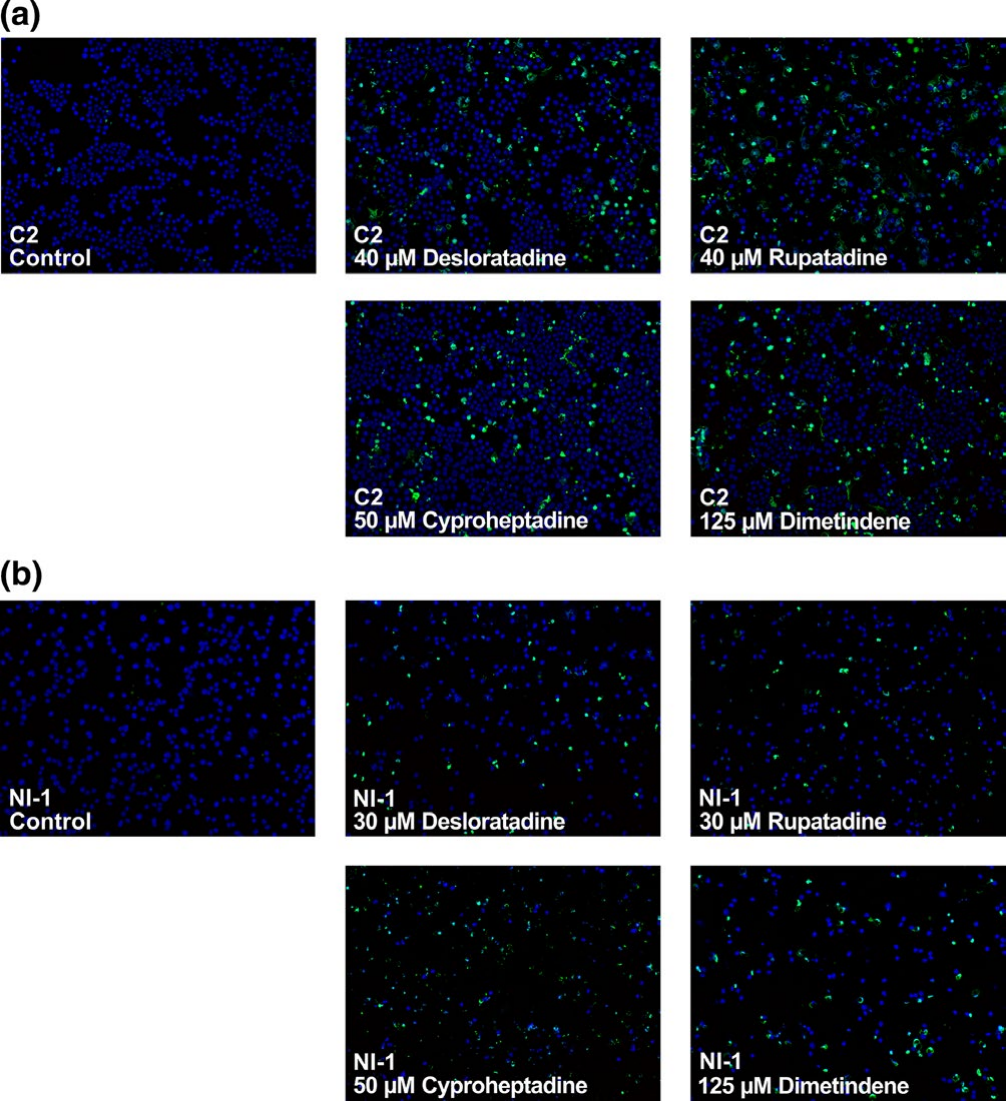
Confirmation of apoptosis‐induction by HR1 antagonists in C2 cells and NI‐1 cells using TUNEL assay. C2 cells (a) and NI‐1 cells (b) were cultured in control medium (Co) or in medium containing HR1 antagonist (as indicated) at 37°C. C2 cells were harvested and analysed after 48 hr. NI‐1 cells were harvested and analysed after 20 hr. TUNEL‐positive (apoptotic) cells are stained in green and TUNEL‐negative/DAPI‐positive cells are stained in blue

We next examined the effects of HR1 antagonists on survival of C2 cells and NI‐1 cells by measuring apoptosis and consecutive necrosis. As determined by light microscopy, desloratadine, rupatadine and cyproheptadine induced apoptosis in C2 cells and NI‐1 cells in a dose‐ and time‐dependent manner (Figure 2). Dimetindene exerted mild effects, whereas diphenhydramine was not able to induce significant apoptosis in both MC lines (Figure 2). ED_50_ ranges are shown in Table 3. Specificity of apoptosis induction in drug‐exposed cells was confirmed by TUNEL assay (Figure 3).

### 3.3 Effects of HR1 antagonists on IgE‐mediated histamine release in NI‐1 cells

**FIGURE 4:**
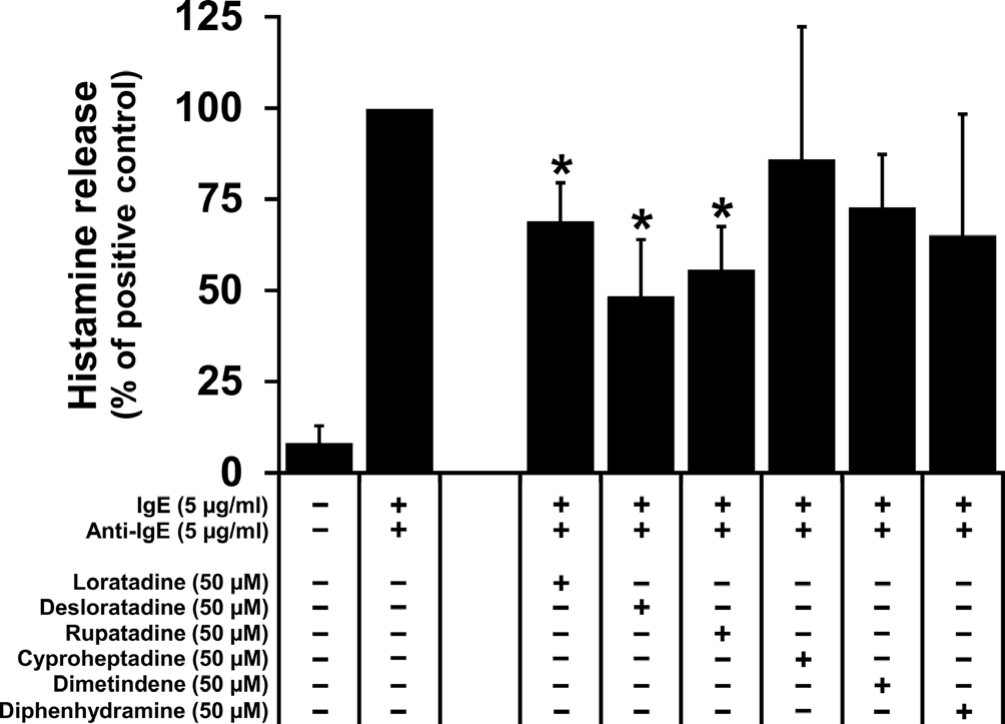
Effects of HR1 antagonists on IgE‐dependent histamine release in NI‐1 cells. NI‐1 cells were pre‐incubated with 5 µg/ml IgE at 37°C for 2 hr, followed by incubation with several HR1 antagonists (as indicated) at 37°C for 60 min. Thereafter, the histamine release was triggered by adding 5 µg/ml anti‐IgE at 37°C for 30 min and afterwards histamine concentrations in the cell‐free supernatants were determined. Histamine release was calculated as percentage of total histamine. Then, the calculated histamine percentages in the ‘IgE + anti‐IgE’ condition were set to 100% and serve as positive control. Results show the percentage of positive control and represent the mean ± *SD* of at least three independent experiments. Asterisk (*): *p* < .05 compared with the ‘IgE + anti‐IgE’ condition (positive control)

To examine whether HR1 antagonists regulate IgE‐dependent histamine release, we performed experiments using NI‐1 cells, known to express a functional and cytokine‐responsive IgE receptor (Bauer et al., 2017; Hadzijusufovic et al., 2012). Loratadine, desloratadine and rupatadine were found to inhibit anti‐IgE‐induced histamine release when applied at a concentration of 50 µM (Figure 4). Desloratadine, a metabolite of loratadine, showed a stronger inhibitory effect on anti‐IgE‐induced histamine release when compared with loratadine and rupatadine. Cyproheptadine, dimetindene and diphenhydramine showed no significant inhibitory effects on histamine release from NI‐1 cells (Figure 4).

### 3.4 Cooperative effects of midostaurin and loratadine on proliferation of canine MC lines

**FIGURE 5:**
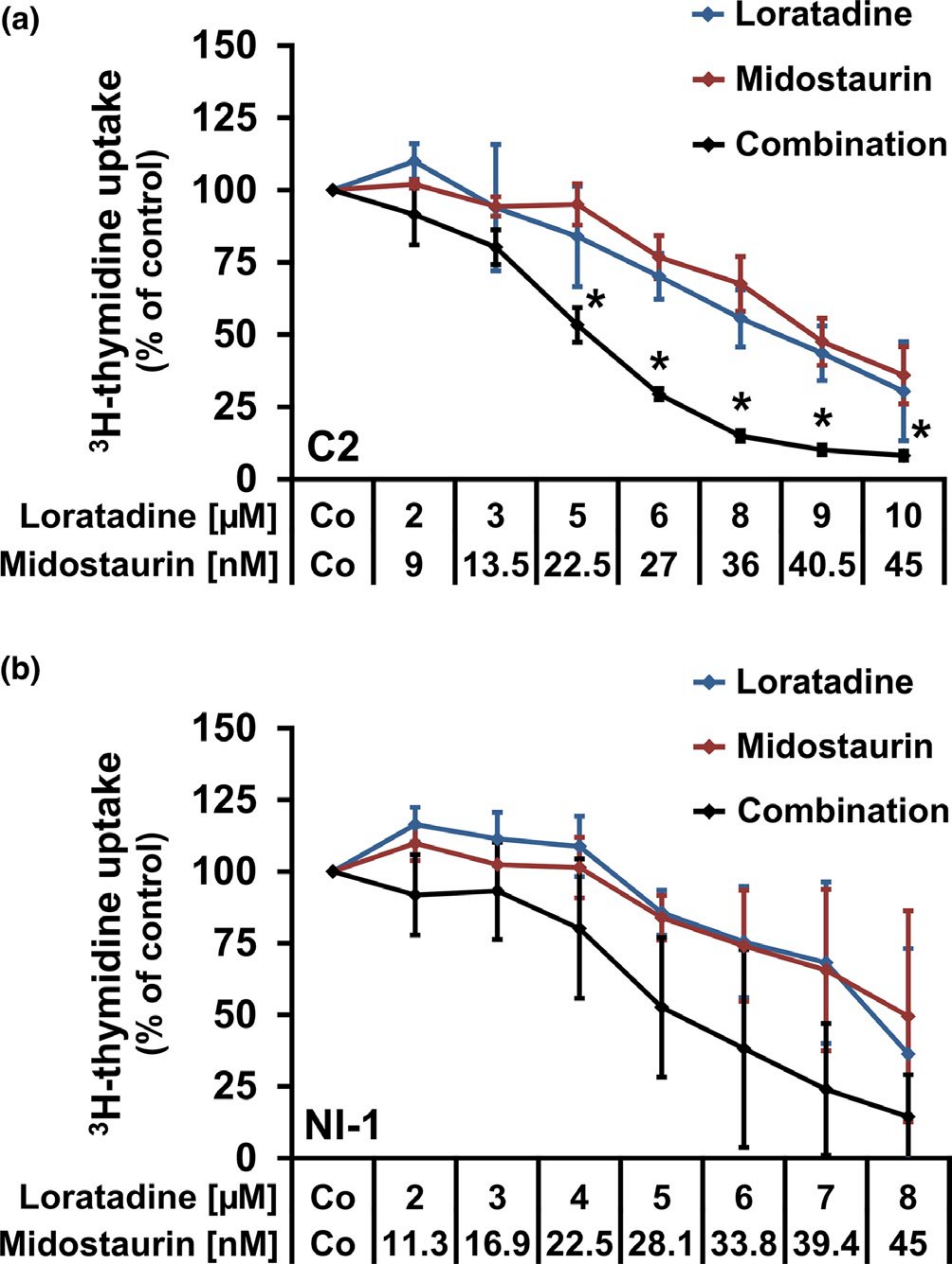
Effects of drug‐combinations on proliferation in canine neoplastic MCs. C2 cells (a) and NI‐1 cells (b) were incubated in control medium (Co) or in medium containing various concentrations of loratadine (blue line), midostaurin (red line) or combinations of these two drugs (black line) at 37°C for 48 hr. Thereafter, ^3^H‐thymidine was added for 16 hr and then the uptake of ^3^H‐thymidine was measured. Results show the ^3^H‐thymidine uptake in percent of control (=100%, Co) and represent the mean ± *SD* of at least three independent experiments. Asterisk (*): *p* < .05 compared with control (Co)

Various TKIs, including masitinib, toceranib and midostaurin (PKC412), exert anti‐proliferative effects against canine (Gleixner et al., 2007; Hahn et al., 2008; London, 2009; London et al., 2009) and human (Gleixner et al., 2013; Gotlib et al., 2016) neoplastic MCs. Masitinib and toceranib are considered to be safe drugs and are effective in decreasing tumour progression in some dogs with high‐grade or non‐resectable MCT (Hahn et al., 2008; London, 2009; London et al., 2009). Midostaurin is well known to suppress the growth of human KIT D816V^+^ MCs *in vitro* and *in vivo* (Gleixner et al., 2013; Gotlib et al., 2016). Since in initial experiments we found that the concentrations of HR1 antagonists required to block MCT cell growth are probably above the tolerable concentration in patients, we were interested to identify drug combinations in which the concentrations of the individual drugs could be reduced to a pharmacologically meaningful range. Therefore, we were interested to learn whether combinations of TKIs and HR1 antagonists can induce growth arrest in canine MCT cells. C2 cells and NI‐1 cells were exposed to various combinations of HR1 antagonists (loratadine, desloratadine, rupatadine, cyproheptadine, dimetindene and diphenhydramine) and KIT‐targeting drugs (masitinib, toceranib, midostaurin). The combination ‘loratadine + midostaurin’ was found to produce cooperative growth‐inhibitory effects (Figure 5). The other drug combinations tested showed no cooperative effects in C2 cells (Figure S3) or in NI‐1 cells (Figure S4).

### 3.5 Effects of midostaurin alone or in combination with desloratadine on IgE‐mediated histamine release in NI‐1 cells

**FIGURE 6:**
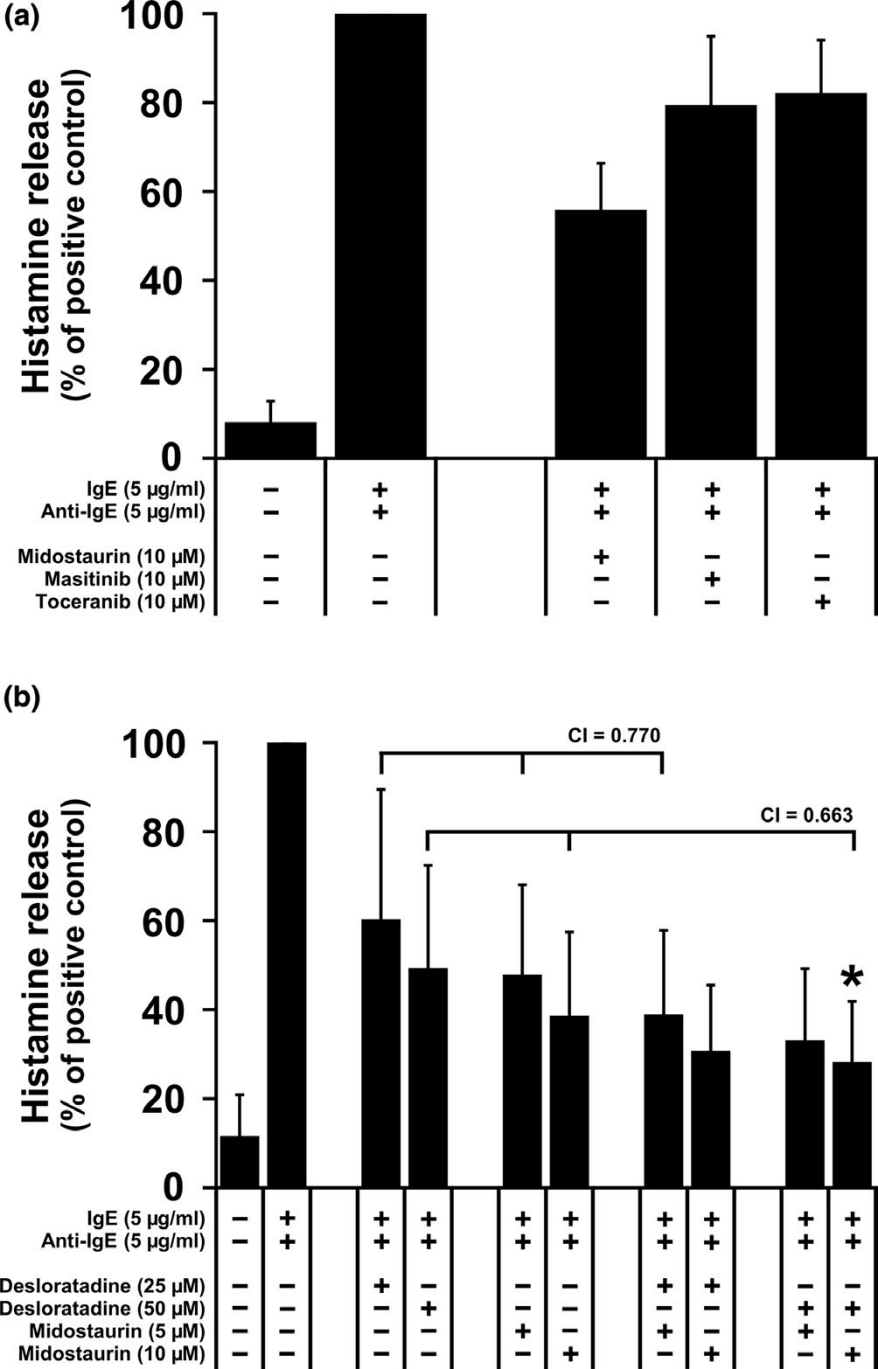
Effects of TKIs and TKI/HR1 antagonist‐combinations on IgE‐dependent histamine release in NI‐1 cells. NI‐1 cells were pre‐incubated with 5 µg/ml IgE at 37°C for 2 hr, followed by an incubation with control medium or medium containing TKIs (depicted in a) or an incubation with control medium, medium containing various concentrations of desloratadine, midostaurin or drug combinations composed of ‘desloratadine + midostaurin’ (depicted in b) at 37°C for 60 min. Thereafter, the histamine release was triggered by adding 5 µg/ml anti‐IgE at 37°C for 30 min and histamine concentrations in the cell‐free supernatants were determined. Histamine release was calculated as percentage of total histamine. Then, the calculated histamine percentages in the ‘IgE + anti‐IgE’ condition were set to 100% and serve as positive control. Results show the percentage of positive control and represent the mean ± *SD* of at least three independent experiments. Asterisk (*): *p* < .05 compared with the ‘IgE + anti‐IgE’ condition (positive control). The combination index (CI) values for the drug‐combinations ‘desloratadine 25 µM + midostaurin 5 µM’ and ‘desloratadine 50 µM + midostaurin 10 µM’ are depicted in b (CI < 1)

Several TKIs, including midostaurin, have been reported to inhibit IgE‐dependent histamine release from human basophils and MCs (Kneidinger et al., 2008; Krauth et al., 2009). In our study, we first asked whether the TKIs masitinib, toceranib and midostaurin alone are able to block IgE‐dependent histamine release in NI‐1 cells. Among the TKIs tested, only midostaurin was able to counteract anti‐IgE‐induced histamine release in NI‐1 cells when applied at a concentration of 10 µM (Figure 6a). Therefore, we combined midostaurin with selected HR antagonists (loratadine and desloratadine) in further IgE‐dependent histamine release experiments. Only the combination ‘desloratadine 50 µM + midostaurin 10 µM’ was found to produce synergistic inhibitory effects on histamine release (Figure 6b). The combination ‘loratadine + midostaurin’ showed no inhibitory effects on histamine release in NI‐1 cells (Figure S5).

## 4 DISCUSSION

Antihistamines are often applied in human and canine MC neoplasms to control mediator‐related clinical symptoms (Arock, Akin, Hermine, & Valent, 2015; Blackwood et al., 2012; Peters & Kovacic, 2009; Sader, Cai, Muller, & Wu, 2017; Thamm & Vail, 2007; Welle et al., 2008; Willmann et al., 2018). However, only few reports on the effects of HR1 antagonists on the proliferation of neoplastic cells are available (Aichberger et al., 2006; Hadzijusufovic et al., 2010; Jangi et al., 2006). We have previously shown that the HR1 antagonists terfenadine and loratadine can suppress the *in vitro* growth of neoplastic MCs (Hadzijusufovic et al., 2010). Due to the removal of terfenadine from the market and the relatively weak anti‐neoplastic effects of loratadine (Hadzijusufovic et al., 2010), we were interested in analysing additional HR1 antagonists for their potential anti‐neoplastic effects. We found that several HR1 antagonists produce growth‐inhibitory and anti‐survival effects in canine neoplastic MCs. In addition, we found that HR1 antagonists loratadine, desloratadine and rupatadine, and the multi‐targeted protein kinase inhibitor midostaurin suppress the IgE‐dependent histamine release from canine neoplastic MCs.

In our ^3^H‐thymidine uptake experiments, several HR1 antagonists showed dose‐dependent growth‐inhibitory effects in canine MC lines and primary MCs. Diphenhydramine was found to exert more potent growth‐inhibitory effects in primary MCs compared with MC lines. We also observed that HR1 antagonists have slightly stronger effects in NI‐1 cells compared with C2 cells. This is consistent with previously reported data, showing NI‐1 cells to be more sensitive to various TKIs than C2 cells (Hadzijusufovic et al., 2012). The reason for this phenomenon remains unknown. A possible explanation for higher sensitivity of NI‐1 cells to anti‐proliferative drugs may be their faster proliferation when compared with C2 cells (Hadzijusufovic et al., 2012). Both cell lines, NI‐1, which were derived from a MC leukaemia and C2, which were derived from solitary MCT and passaged in nude mice, represent rather immature neoplastic MCs (DeVinney & Gold, 1990). NI‐1 cells and C2 cells are blast cells from a morphologically point of view; both cells have a prominent, round nucleus and the cytoplasm shows numerous vacuoles and very few metachromatic granules.

Previously, we have shown that terfenadine and loratadine not only inhibit proliferation, but also reduce survival of neoplastic MCs (Hadzijusufovic et al., 2010). Using morphological assessment and TUNEL assay, we found that desloratadine, rupatadine and cyproheptadine induced significant dose‐ and time‐dependent apoptosis in both C2 cells and NI‐cells. These data suggest that the HR1 antagonists tested do not exert toxic effects, but rather initiate apoptosis in canine neoplastic MC lines. In contrast to our experiments on cell proliferation, higher concentrations were necessary to achieve a comparable effect on apoptosis (e.g. incubation with 50 µM of desloratadine results in a total inhibition of proliferation in C2 cells, whereas in our apoptosis‐induction experiments approximately 50% of cells remain viable when the same concentration is applied, at least considering morphological criteria). This is consistent with studies on human melanoma and hepatocellular carcinoma, in which diphenhydramine and cyproheptadine (applied in a range of 40–100 µM) induced apoptosis, but no toxic necrosis (Feng et al., 2015; Or et al., 2016).

Our data suggest that pharmacologically active concentrations needed for inhibiting MC proliferation and survival are higher than can be achieved *in vivo* in mastocytoma patients, even if applied in up to four‐time higher doses, which are well tolerated by humans (Gimenez‐Arnau et al., 2009; Gupta et al., 2002; Powell et al., 2007; Siebenhaar et al., 2009; Zuberbier, 2009; Zuberbier et al., 1996). Therefore, further efforts to identify more effective and less toxic drug derivatives are needed. However, it seems that the observed effects are at least in part mediated through HR1‐binding, as several chemically diverse HR1 antagonists showed anti‐neoplastic effects, whereas none of the HR2 antagonists applied showed effects on neoplastic cells in previous studies (Aichberger et al., 2006; Hadzijusufovic et al., 2010). Nevertheless, as not all HR1 antagonists exhibit the same anti‐proliferative and pro‐apoptotic effects in C2 and NI‐1 cells, it remains unclear if the effects observed in this study, are indeed all mediated via HR1. Potential other targets of HR1 antagonists might be intracellular histamine receptors, such as cytochrome P450 isoenzymes (Dy & Schneider, 2004). Certain cytochrome P450 isoenzymes have been suggested to play a role as targets of loratadine and terfenadine by mediating growth‐inhibitory effects in chronic myeloid leukaemia cells (Aichberger et al., 2006). Previous studies have also shown that histamine and histamine‐metabolizing enzymes, both known to interact with cytochrome P450 isoenzymes, regulate the growth and survival of various tumour cells (Darvas et al., 2003; Falus et al., 2001; Malaviya & Uckun, 2000; Radvány et al., 2000; Rivera et al., 2000). Some of these interactions may be associated with autocrine or intercrine growth regulation in neoplastic cells (Darvas et al., 2003; Falus et al., 2001; Malaviya & Uckun, 2000; Radvány et al., 2000; Rivera et al., 2000). Since MCs produce and contain substantial amounts of histamine it also might be that certain HR1 antagonists are able to disrupt autocrine or intercrine pathways involving histamine and histamine‐binding molecules in neoplastic MCs. Further studies are required to learn about the precise mechanisms of action of HR1 antagonists on intracellular pathways and growth of canine neoplastic MCs.

So far, only the HR1 antagonists loratadine and rupatadine have been tested for their effects on IgE receptor‐dependent histamine release from canine non‐neoplastic skin MCs and potent effects were observed with a concentration of 30 µM for both drugs (Queralt, Brazís, Merlos, de Mora, & Puigdemont, 2000). However, no studies using canine neoplastic MCs have been reported so far. In our study, desloratadine, rupatadine and loratadine were able to suppress anti‐IgE‐induced histamine release in neoplastic MCs. A concentration of 50 µM was needed to obtain similar inhibitory effects as seen with 30 µM in non‐neoplastic skin MCs. It seems that normal MCs are more sensitive to the effects of HR1 antagonists than neoplastic MCs. An explanation for this phenomenon may be that neoplastic MCs are more immature cells and therefore express lower amounts of histamine receptors or a different (less responsive) network of downstream effector molecules. However, the exact mechanisms underlying the response of canine MCT cells to certain HR1 antagonists remains unknown.

Since HR1 antagonists are commonly used in parallel with anti‐neoplastic drugs during MCT‐treatment (Blackwood et al., 2012; Peters & Kovacic, 2009; Welle et al., 2008), we asked whether combining HR1 antagonists with TKIs would result in cooperative effects. In our study, the drug‐combination ‘loratadine + midostaurin’ was the most potent combination resulting in cooperative growth‐inhibitory effects in C2 and NI‐1 cells, yet the concentrations of HR1 antagonists required to achieve these effects were relatively high and above the usually administered drug concentrations. The other combinations tested showed no cooperative effects.

Since the TKIs midostaurin and dasatinib were reported to inhibit IgE‐dependent histamine release from human basophils and MCs (Kneidinger et al., 2008; Krauth et al., 2009), we asked whether TKIs that are already in use for treatment of canine or human neoplastic MC disorders are also able to block IgE‐dependent histamine release in canine neoplastic MCs. For histamine release experiments, all TKIs were applied for only one hour, therefore, we used higher concentrations of these TKIs compared to proliferation experiments where these drugs were applied for 24 or 48 hr (Hadzijusufovic et al., 2012). In these experiments, midostaurin was found to counteract anti‐IgE‐induced histamine release at relatively high concentrations, exceeding those that are effective in human MCs (Krauth et al., 2009). Masitinib and toceranib showed no effects on IgE‐mediated histamine release. We also combined midostaurin with loratadine and its active metabolite desloratadine. However, only the combination ‘desloratadine + midostaurin’ produced synergistic inhibitory effects on IgE‐dependent histamine release in NI‐1 cells. Since midostaurin not only blocks mediator release but also inhibits proliferation of canine neoplastic MCs (Gotlib et al., 2016; Hadzijusufovic et al., 2012), this agent may be a promising drug for the treatment of canine mastocytoma.

In summary, our data show that the HR1 antagonists desloratadine, rupatadine and cyproheptadine reduce proliferation and survival of canine MC lines more effectively than dimetindene and diphenhydramine. In contrast, diphenhydramine was found to exert more potent growth‐inhibitory effects in primary neoplastic MCs when compared to the other HR1 antagonists tested. Why the HR1 antagonists exert different biologic activities in MC lines and in primary MCs remains unknown. Moreover, we found that loratadine, desloratadine and rupatadine inhibit IgE‐dependent histamine release in NI‐1 cells. However, concentrations needed for these effects are higher than those usually applied in patients. Whether more potent derivatives combining anti‐mediator with growth‐inhibitory effects can be developed, remains to be elucidated.

## CONFLICT OF INTEREST

The authors declare that they have no conflict of interest. Conflict of interest unrelated to this study: PV received honoraria from Novartis, Blueprint, Deciphera and Incyte.

## AUTHOR CONTRIBUTION

**Susanne Gamperl:** Conceptualization; Data curation; Formal analysis; Funding acquisition; Investigation; Methodology; Project administration; Software; Visualization; Writing‐original draft; Writing‐review & editing. **Gabriele Stefanzl:** Data curation; Methodology; Software; Validation; Visualization; Writing‐review & editing. **Michael Willmann:** Formal analysis; Investigation; Project administration; Resources; Validation; Writing‐original draft; Writing‐review & editing. **Peter Valent:** Data curation; Formal analysis; Funding acquisition; Resources; Supervision; Validation; Writing‐review & editing. **Emir Hadzijusufovic:** Conceptualization; Formal analysis; Funding acquisition; Investigation; Project administration; Resources; Software; Supervision; Validation; Writing‐original draft; Writing‐review & editing.

### PEER REVIEW

The peer review history for this article is available at https://publons.com/publon/10.1002/vms3.336.

## Supporting information

Supplementary Material

## Data Availability

Please contact the corresponding author for all reasonable data requests.
