# Supplementary material for: In vitro effects of histamine receptor 1 antagonists on proliferation and histamine release in canine neoplastic mast cells
Source: Vet Med Sci. 2020 Sep 13;7(1):57–68. doi: 10.1002/vms3.336 (PMC7840218; doi:10.1002/vms3.336)
Supplement: Supplementary file 1 — Supplementary Material [file VMS3-7-57-s001.docx]

**Supplementary Material to Manuscript:**

***In vitro* effects of histamine receptor 1 antagonists on proliferation and histamine release in**

**canine neoplastic mast cells**

**Supplementary Figures**

***
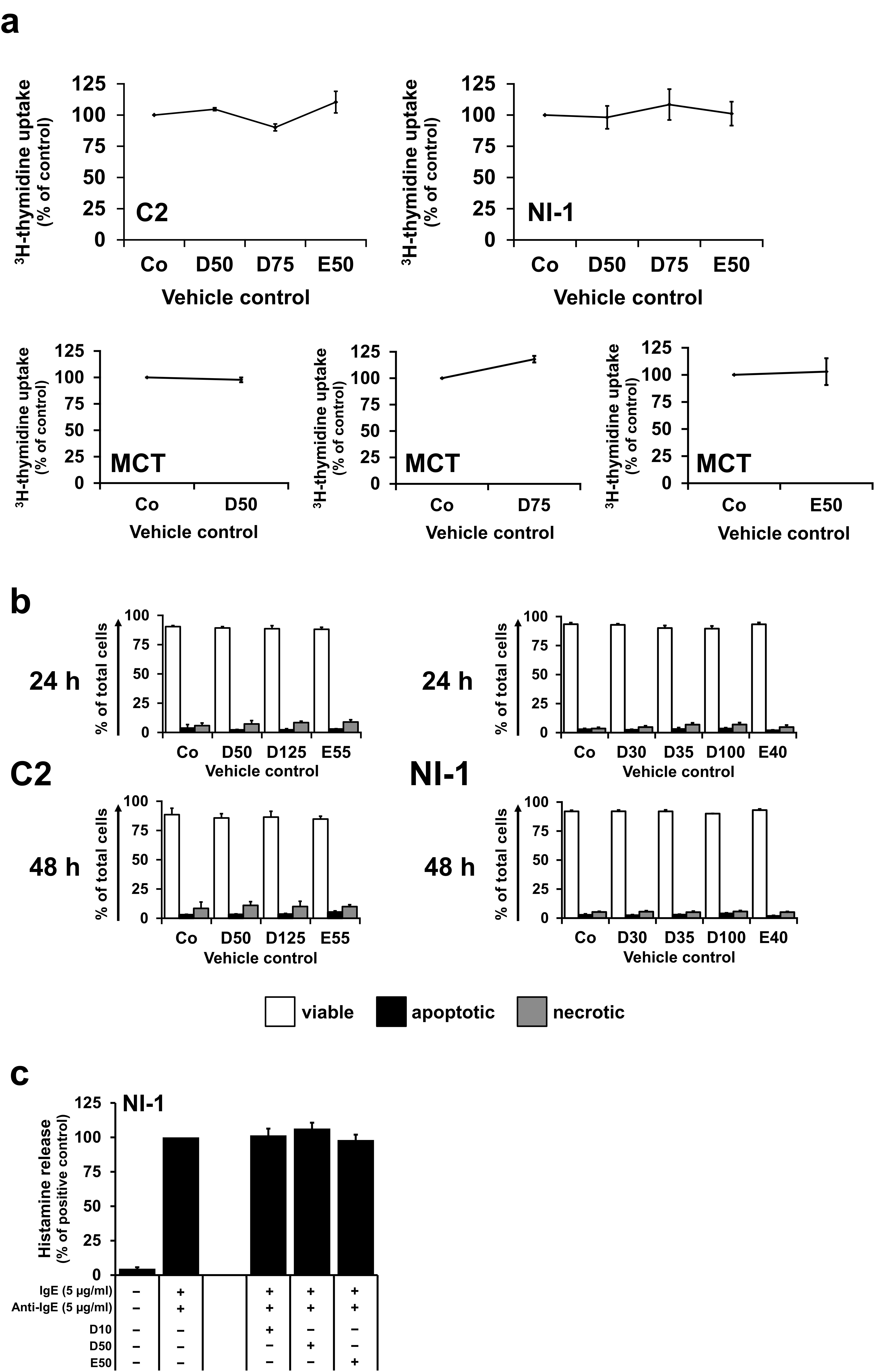
***

***Fig. S1***

***Fig. S1. Effects of vehicle controls in canine neoplastic mast cells.***

**(a)** C2, NI-1 cells and primary cells isolated from MCT #3 were incubated in control medium (Co) or in medium containing DMSO (D) or ethanol (E) in concentrations corresponding to the amount of vehicle substance in the highest drug concentrations at 37°C for 48 hours. D50 corresponds to 50 µM desloratadine or rupatadine, D75 to 75 µM dimetindene or diphenhydramine, and E50 to 50 µM cyproheptadine. Thereafter, ^3^H-thymidine was added for 16 hours and then the uptake of ^3^H-thymidine was measured. Results show ^3^H-thymidine uptake in percent of control (=100%, Co) and represent the mean±SD of triplicates.

**(b)** C2 and NI-1 cells were incubated in control medium (Co) or in medium containing DMSO (D) or ethanol (E) in concentrations corresponding to the amount of vehicle substance in the highest drug concentrations at 37°C for 24 hours (upper panels) or 48 hours (lower panels). In C2 cells, D50 corresponds to 50 µM desloratadine or rupatadine, D125 to 125 µM dimetindene or diphenhydramine, and E55 to 55 µM cyproheptadine. In NI-1 cells, D30 corresponds to 30 µM desloratadine, D35 to 35 µM rupatadine, D100 to 100 µM dimetindene or diphenhydramine, and E40 to 40 µM cyproheptadine. Thereafter, the cells were stained using the Hematek® Stain Pak (Modified Wright´s Stain) and the numbers of viable, apoptotic and necrotic cells were counted using light microscopy. Results show the percentage (%) of viable (white open bars), apoptotic (black filled bars) and necrotic (grey filled bars) cells relative to the total cell number. Results represent the mean±SD of at least three independent experiments.

**(c)** NI-1 cells were preincubated with 5 µg/ml IgE at 37°C for 2 hours, followed by an incubation with control medium or medium containing DMSO (D) or ethanol (E) in concentrations corresponding to the amount of vehicle substance in the highest drug concentrations at 37°C for 60 minutes. D10 corresponds to 10 µM midostaurin, masitinib or toceranib, D50 to 50 µM loratadine, desloratadine, rupatadine, dimetindene, or diphenhydramine, E50 to 50 µM cyproheptadine. Thereafter, the histamine release was triggered by adding 5 µg/ml anti-IgE at 37°C for 30 minutes and histamine concentrations in the cell-free supernatants were determined. Histamine release was calculated as percentage of total histamine. Then, the calculated histamine percentages in the `IgE+anti-IgE´ condition were set to 100% and serve as positive control. Results show the percentage of positive control and represent the mean±SD of triplicates.

***
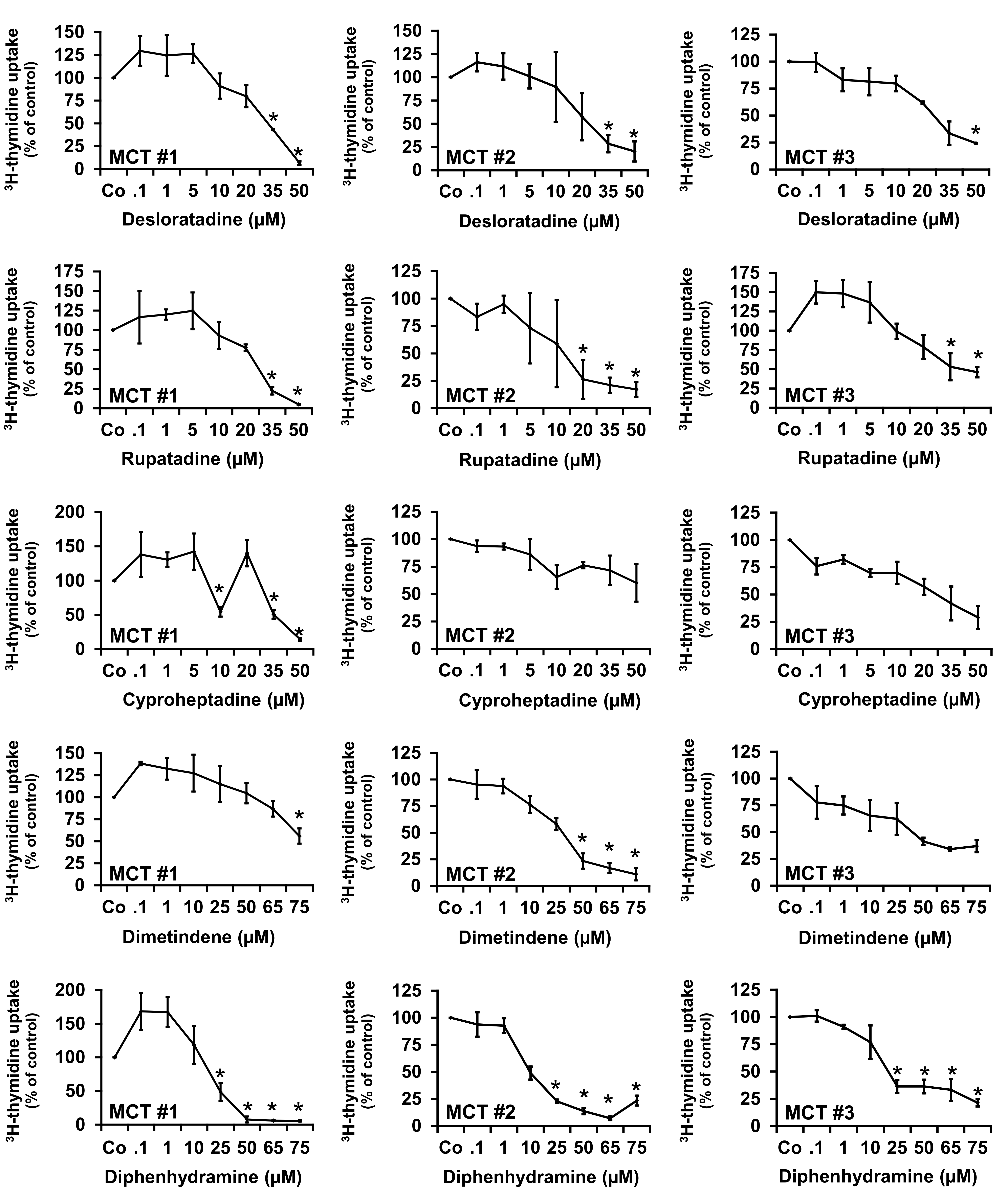
***

***Fig. S2. Effects of HR1 antagonists on proliferation in primary MCT cells.***

Primary mast cells from three individual patients (MCT #1-3) were incubated in control medium (Co) or in medium containing increasing concentrations of various HR1 antagonists (as indicated) at 37°C for 48 hours. Thereafter, ^3^H-thymidine was added for 16 hours and then the uptake of ^3^H-thymidine was measured. Results show the ^3^H-thymidine uptake in percent of control (=100%, Co) and represent the mean±SD of triplicates. Asterisk (*): *P*<0.05 compared with control (Co).

**
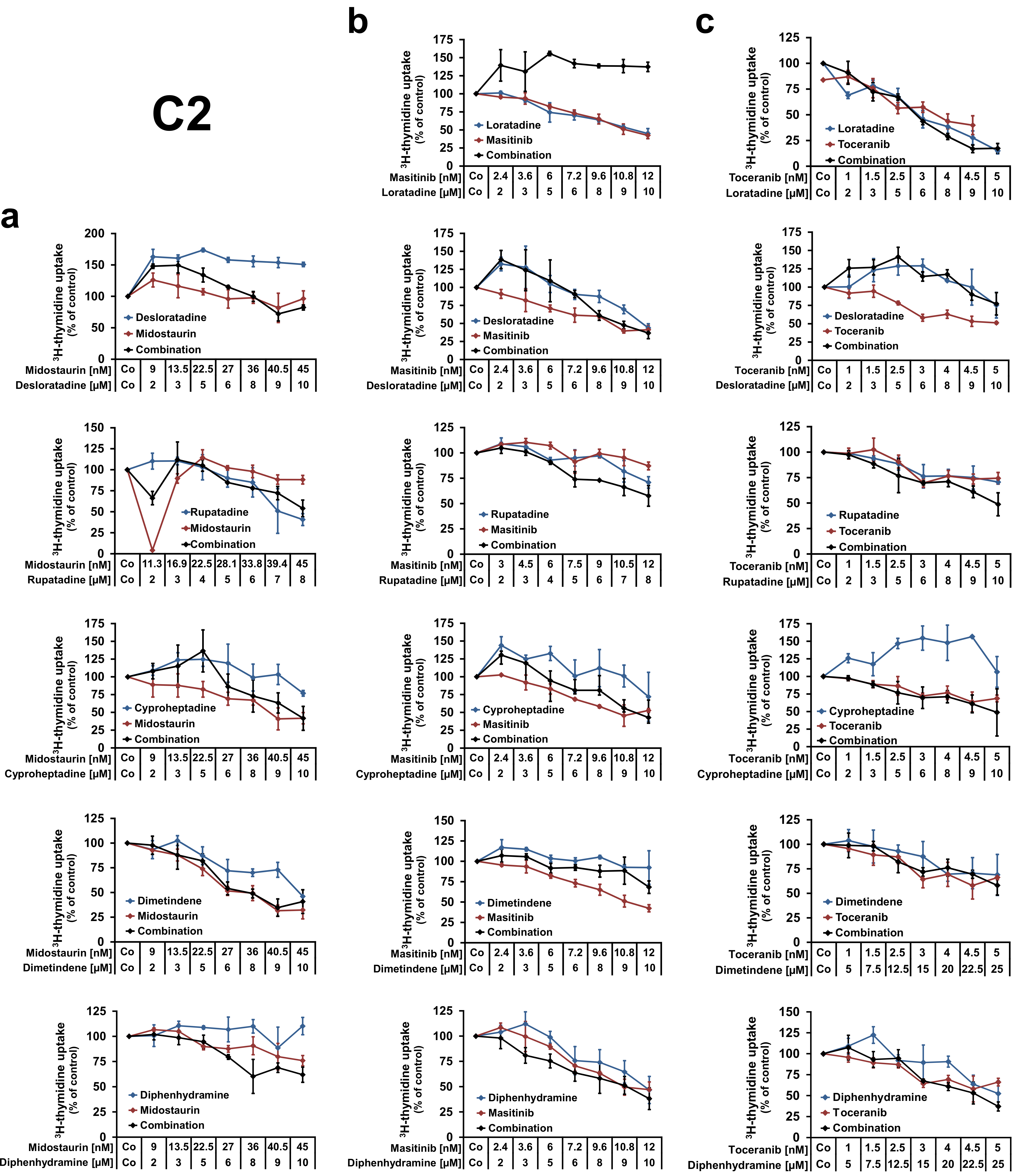
**

***Fig. S3. Effects of drug-combinations on proliferation in C2 cells.***

C2 cells were incubated in control medium (Co) or in medium containing increasing concentrations of one TKI (**a**: midostaurin, **b**: masitinib, **c**: toceranib) or various HR1 antagonists (as indicated), or combinations of two drugs (as indicated) at 37°C for 48 hours. TKIs are represented by red lines, HR1 antagonists by blue lines, and drug-combinations by black lines. Thereafter, ^3^H-thymidine was added for 16 hours and then the uptake of ^3^H-thymidine was measured. Results show the ^3^H-thymidine uptake in percent of control (=100%, Co) and represent the mean±SD of triplicates.

**
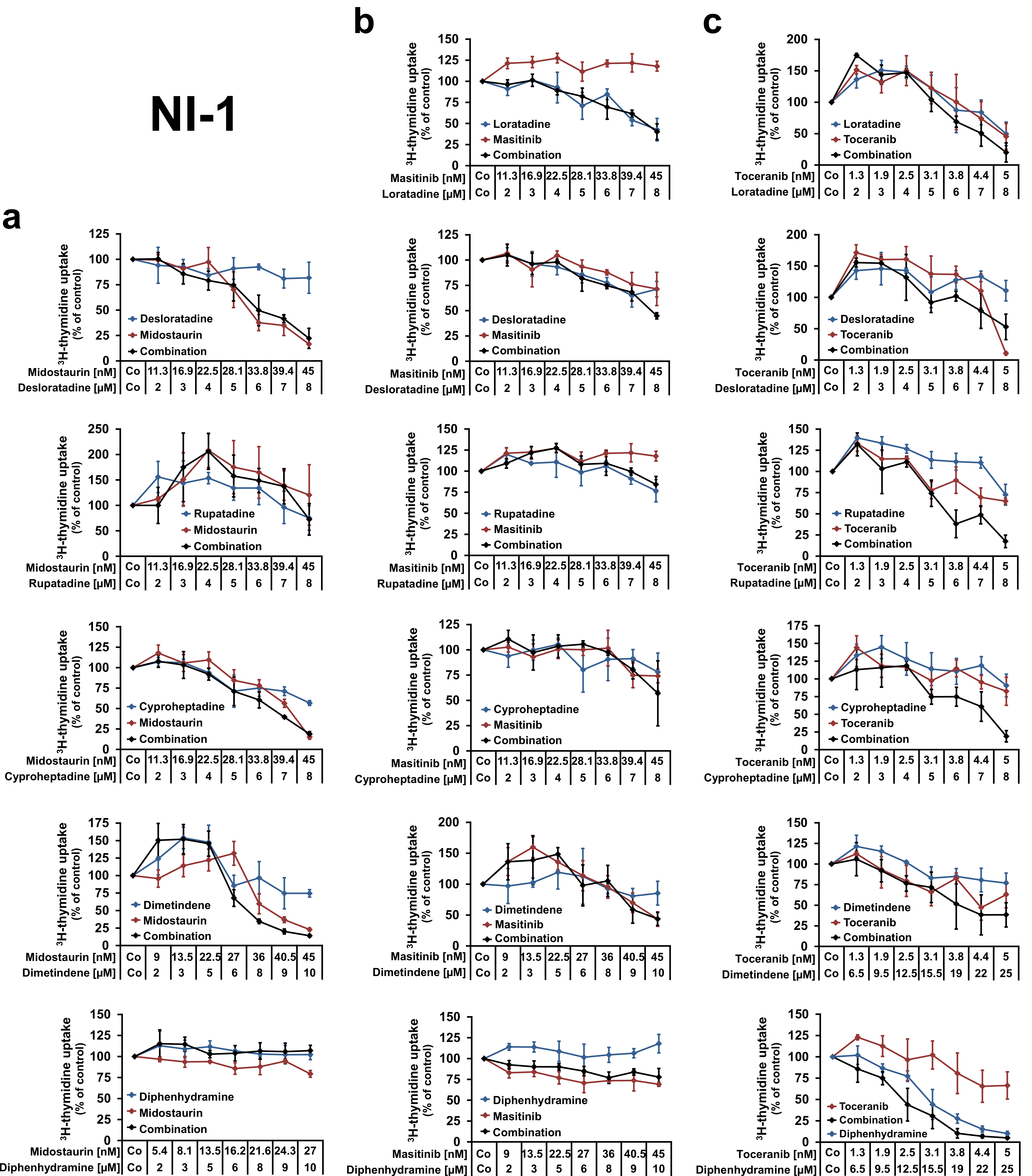
**

***Fig. S4. Effects of drug-combinations on proliferation in NI-1 cells.***

NI-1 cells were incubated in control medium (Co) or in medium containing increasing concentrations of one TKI (**a**: midostaurin, **b**: masitinib, **c**: toceranib) or various HR1 antagonists (as indicated), or combinations of two drugs (as indicated) at 37°C for 48 hours. TKIs are represented by red lines, HR1 antagonists by blue lines, and drug-combinations by black lines. Thereafter, ^3^H-thymidine was added for 16 hours and then the uptake of ^3^H-thymidine was measured. Results show the ^3^H-thymidine uptake in percent of control (=100%, Co) and represent the mean±SD of triplicates.

**
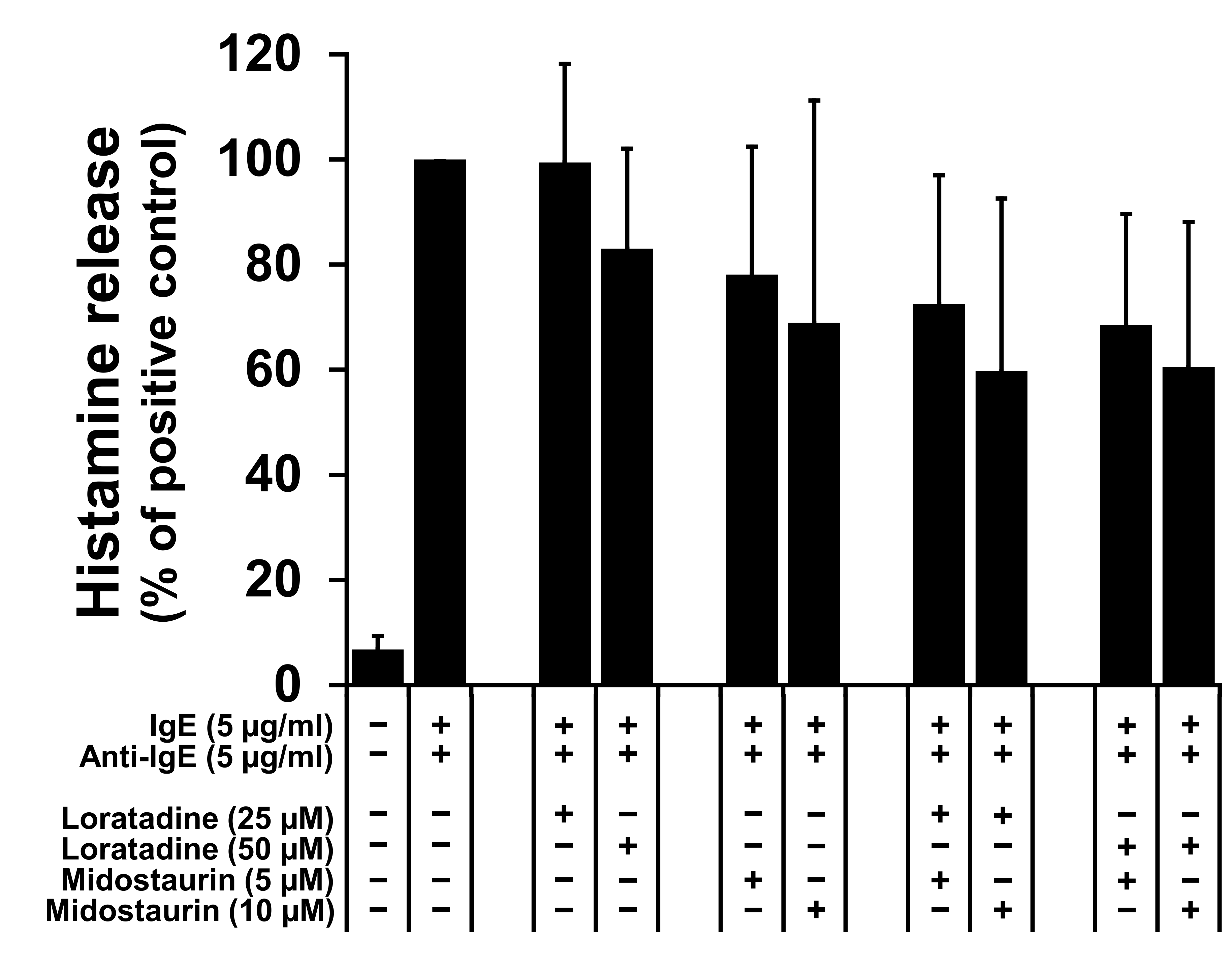
**

***Fig. S5. Effects of loratadine and midostaurin alone or of combinations of both drugs on IgE-dependent histamine release in NI-1 cells.***

NI-1 cells were preincubated with 5 µg/ml IgE at 37°C for 2 hours, followed by an incubation with control medium or medium containing various concentrations of loratadine, midostaurin or drug combinations composed of `loratadine+midostaurin´ at 37°C for 60 minutes. Thereafter, the histamine release was triggered by adding 5 µg/ml anti-IgE at 37°C for 30 minutes and histamine concentrations in the cell-free supernatants were determined. Histamine release was calculated as percentage of total histamine. Then, the calculated histamine percentages in the `IgE+anti-IgE´ condition were set to 100% and serve as positive control. Results show the percentage of positive control and represent the mean±SD of at least three independent experiments.
